# Rapid growth of primary pulmonary meningioma with hemoptysis

**DOI:** 10.1093/omcr/omae051

**Published:** 2024-05-20

**Authors:** Rena Tamenaga, Yohei Kawaguchi, Mariko Kogami, Taro Kufukihara, Reimi Mizushima, Yukihisa Takeda, Yusuke Watanabe, Kinya Furukawa, Hiroyuki Nakamura, Kazutetsu Aoshiba

**Affiliations:** Department of Respiratory Medicine, Tokyo Medical University Ibaraki Medical Center, Ibaraki, Japan; Department of Respiratory Medicine, Tokyo Medical University Hospital, Tokyo, Japan; Department of Thoracic Surgery, Tokyo Medical University Ibaraki Medical Center, Ibaraki, Japan; Department of Thoracic Surgery, Tokyo Medical University Hospital, Tokyo, Japan; Department of Respiratory Medicine, Tokyo Medical University Ibaraki Medical Center, Ibaraki, Japan; Department of Respiratory Medicine, Tokyo Medical University Hospital, Tokyo, Japan; Department of Respiratory Medicine, Tokyo Medical University Ibaraki Medical Center, Ibaraki, Japan; Department of Respiratory Medicine, Tokyo Medical University Hospital, Tokyo, Japan; Department of Respiratory Medicine, Tokyo Medical University Ibaraki Medical Center, Ibaraki, Japan; Department of Respiratory Medicine, Tokyo Medical University Hospital, Tokyo, Japan; Department of Respiratory Medicine, Tokyo Medical University Ibaraki Medical Center, Ibaraki, Japan; Department of Respiratory Medicine, Tokyo Medical University Ibaraki Medical Center, Ibaraki, Japan; Department of Infection Prevention and Control, Tokyo Medical University Hospital, Tokyo, Japan; Department of Thoracic Surgery, Tokyo Medical University Hospital, Tokyo, Japan; The Fraternity Memorial Hospital, Tokyo, Japan; Department of Respiratory Medicine, Tokyo Medical University Ibaraki Medical Center, Ibaraki, Japan; Department of Respiratory Medicine, Tokyo Medical University Ibaraki Medical Center, Ibaraki, Japan

**Keywords:** meningioma, lung tumor, hemorrhage, hemoptysis

## Abstract

While lung cancer is the predominant neoplasm causing hemoptysis, rare benign neoplasms can also be associated with hemoptysis. A 60-year-old woman presented with cough and hemoptysis. Chest computed tomography revealed an oval-shaped, well-circumscribed solitary mass (10 cm in size) in the right lower lobe, which had grown rapidly over the past year. The presence of intramass air bubbles and a surrounding halo of ground-glass opacities suggested the hemorrhagic rupture of a circumscribed hematoma into the surrounding lung tissue. Subsequent right lower lobectomy revealed a well-demarcated hematoma; its wall consisted of nonatypical spindle tumor cells, which were histologically diagnosed as meningioma. No meningioma was observed in the central nervous system, leading to the diagnosis of primary pulmonary meningioma. This case highlights PPM as a rare benign tumor (World Health Organization grade 1) capable of rapid development due to intratumoral hemorrhage, presenting with hemoptysis.

## INTRODUCTION

Hemoptysis is a commonly encountered presentation in respiratory medicine practice, with its causes including neoplasms, tuberculosis, mycetoma, and bronchiectasis [1]. While lung cancer remains the predominant neoplasm associated with hemoptysis, rare benign neoplasms, such as hamartoma and pulmonary sclerosing pneumocytoma [2], are linked to infrequent instances of hemoptysis. This report presents the case of a patient with benign primary pulmonary meningioma (PPM, World Health Organization [WHO] grade 1) that developed rapidly due to intratumoral hemorrhage, presenting with hemoptysis.

## CASE REPORT

A 60-year-old woman was admitted to our hospital with a two-day history of cough and hemoptysis. She had a medical history of well-controlled diabetes mellitus, hypertension, and hyperlipidemia, with no history of cigarette smoking. On examination, she was afebrile and normoxemic (SpO_2_, 97%), with no wheezes, lung crackles, or abnormal heart sounds on auscultation.

Blood tests revealed mild leukocytosis (11 700 cells/μl with 80.2% neutrophils, 1.2% eosinophils, and 13.8% lymphocytes) with slightly elevated levels of C-reactive protein (2.22 mg/dl) and hemoglobin A1c (6.5%). Liver and renal function tests were within the normal range. Serum tumor markers, including carcinoembryonic antigen, cytokeratin-19 fragment, and pro-gastrin-releasing peptide, were normal. Serum levels of β-D-glucan, aspergillus antigen, and cryptococcus antigen titer were also normal. The interferon-gamma releasing assay (T-spot.^®^TB test) yielded a negative result.

**Figure 1:**
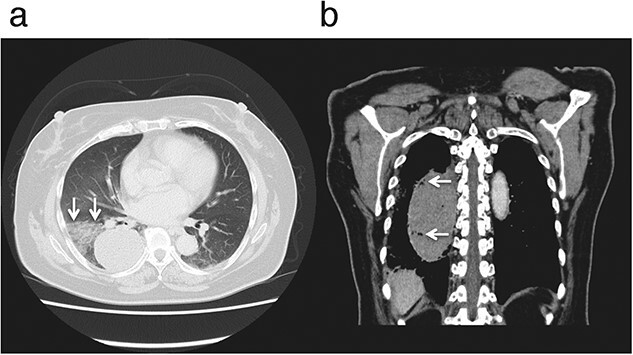
Chest contrast-enhanced computed tomography images of a hemoptysis patient showing a nonenhanced, oval-shaped, well-circumscribed solitary mass in the right lower lobe. (**a**) Transverse view of the lung window image reveals a surrounding halo of ground-glass opacities (*arrows*). (**b**) Coronal view of the mediastinal window image displays intramass air bubbles (*arrows*). Both findings indicate the rupture of a hematoma into the lung tissue.

Chest X-ray and contrast-enhanced computed tomography (CT) revealed a non-enhanced, oval-shaped, well-circumscribed solitary mass measuring 5 × 6 × 10 cm in the right lower lobe (Fig. 1). The presence of intramass air bubbles and a surrounding halo of ground-glass opacities indicated the hemorrhagic rupture of a circumscribed hematoma into the surrounding lung tissue. A 1-cm nodule in the right pulmonary hilum was retrospectively detected in her chest X-ray taken one year earlier. Positron emission tomography/CT imaging showed no pathological accumulation of 18-fluoro-2-deoxy-D-glucose was observed in the mass.

Bronchoscopy performed on the seventh day of admission revealed active bleeding from the superior segmental bronchus (B6) of the right lower lobe. However, no tumorous lesions were found in the visible area of the airway. The cytology and bacterial culture of the bronchial lavage fluid had negative results. Biopsy of the tumor lesion was not performed because the bronchoscope forceps did not hit the mass. Our clinical differential diagnoses included lung cancer, rare benign hemorrhagic neoplasms, lung abscess, mycetoma, and congenital malformations, including congenital cystic adenomatoid malformation and bronchopulmonary sequestration.

**Figure 2:**
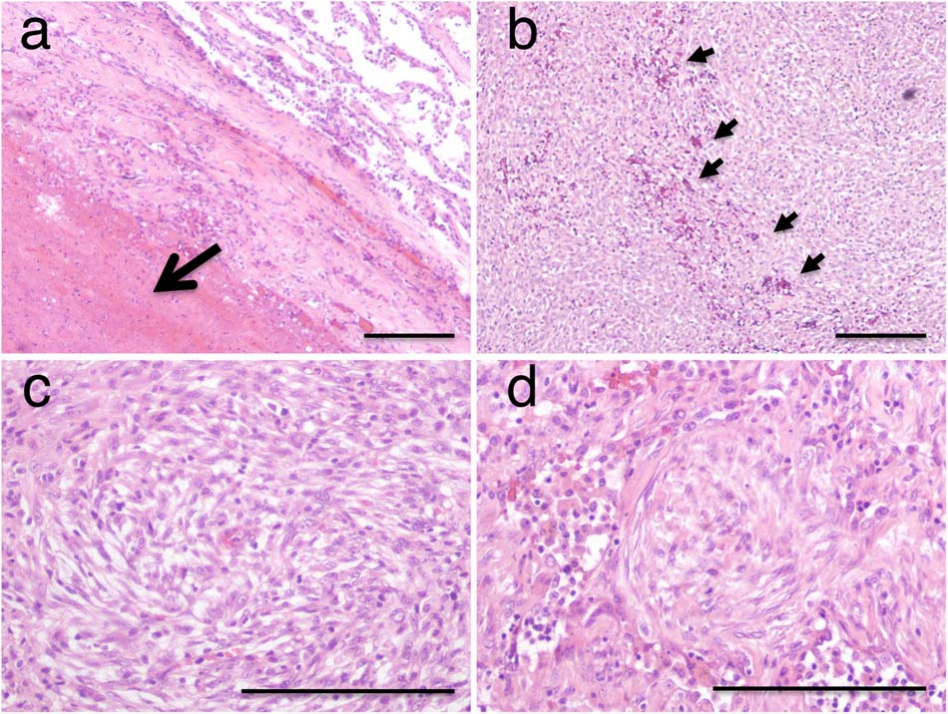
Histological features of the surgically resected mass in low-power (**a** and **b**) and high-power (**c** and **d**) magnifications of a hematoxylin and eosin-stained specimen. The interior of the mass consisted of a fresh hematoma filled with red blood cells (*large arrow*) (**a**). The mass wall comprised spindle tumor cells with a sheet or whorl pattern (**c** and **d**) interspersed with numerous thin-walled vasculatures (*small arrows*) (**b**). Nuclear atypia, mitotic figures, or necrosis was not observed in the tumor cells (**c** and **d**). Scale bar: 100 μm.

**Figure 3:**
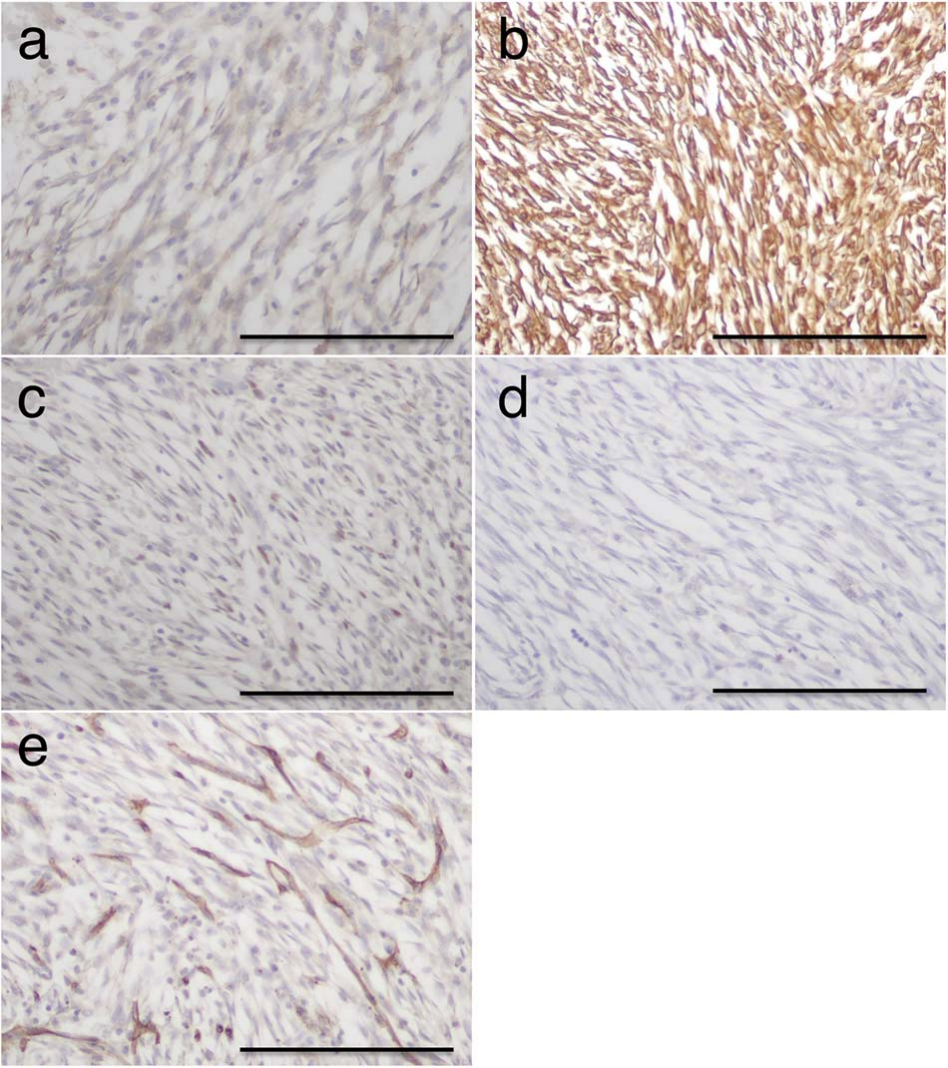
Immunohistochemical staining of tumor cells. The tumor cells tested positive for epithelial membrane antigen (**a**), vimentin (**b**), and progesterone receptor (**c**), but negative for S100 (**d**) and CD34 (**e**). Immunoposive cells (**e**) are vascular cells. Scale bar: 100 μm.

The patient subsequently underwent a right lower lobectomy, revealing a well-demarcated hematoma (Fig. 2). Its wall consisted of nonatypical spindle tumor cells arranged in a sheet or whorl pattern interspersed with numerous vasculatures. Moreover, immunohistochemical analysis showed positive staining for epithelial membrane antigen, vimentin, and progesterone receptor, while S100 and CD34 were negative (Fig. 3). The histological diagnosis was meningioma [3, 4]. Nuclear atypia, mitotic figures, or necrosis was not observed in the tumor cells (Fig. 2). The MIB-1 index was 10%–20%. According to the 2021 WHO classification of brain tumors [5], the tumor was classified as grade 1 based on the histological examination. Magnetic resonance imaging showed no intracranial or spinal meningioma. Based on these findings, the patient was diagnosed with PPM and did not present with recurrence or metastasis two years after surgery.

## DISCUSSION

The PPM is an exceptionally rare ectopic meningioma that may originate from minute pulmonary menigothelial-like nodules or pluripotent subpleural mesenchyme [4, 6]. A limited number of cases, approximately 70, have been documented in the English literature [6, 7]. Radiologically, PPM typically manifests as a well-defined solid nodule [6, 7]. Distinguishing PPM from other lung tumors based solely on radiological findings is challenging without pathological and immunohistochemical examination. Diagnosis of PPM relies on the presence of a tumor in the lungs, thereby concurrently ruling out meningioma in the central nervous system. In most PPM cases, an asymptomatic pulmonary nodule may emerge, with only three reported cases with hemoptysis [7, 8]. Notably, complications related to hemorrhage, occurring in approximately 2.2% of patients with intracranial meningioma, are often attributed to the rupture of defective intratumoral blood vessels and overstretched veins surfacing the growing tumor [9]. In cases of lung cancer, hemoptysis can occur through various mechanisms, including neovascularization in and around the tumor, invasion of vascular structures by the tumor, exfoliation of the tumor surface, and tumor necrosis [10]. In the current PPM case, the tumor tissues exhibited numerous thin-walled vasculatures within the tumor stroma, without evident vascular invasion or tumor cell exfoliation. This led to the suspicion that a high degree of neovascularization likely contributed to the etiology of hemoptysis in our PPM case.

While primary lung cancer and metastatic tumors represent the most commonly encountered neoplasms for clinicians, benign lung tumors are rare clinical entities. These tumors include bronchiolar adenoma/ciliated muconodular papillary tumor, sclerosing pneumocytoma, alveolar adenoma, bronchial papillomas, pulmonary hamartoma, and pulmonary chondroma [11]. These benign tumors typically exhibit a slow growth rate, and they are symptomatic. Our case stands out due to the exceptional characteristics of benign tumor such as rapid growth over one year before its discovery, which is likely attributed to intratumoral hemorrhage. In this case, hemoptysis could have been caused by the direct rupture of the hematoma, rapid growth-related bronchial luminal mucosal erosion and submucosal vascular irritation or invasion, and rapid tumor growth-related bronchial irritation resulting in provocative cough associated with hemorrhage. In conclusion, PPM is a rare benign tumor with the potential for swift development due to intratumoral hemorrhage, leading to its manifestation with hemoptysis.
